# Does Cincho-Quinine Embody All the Properties of the Cinchona Bark?

**Published:** 1878-04-20

**Authors:** 


### DOES CINCHO-QUININE EMBODY ALL THE PROPERTIES OF THE CINCHONA BARK?

It is well known that the manufacturers of the cincho-quinine claim that it contains all the alkaloids of the Peruvian bark, and is equally effectual, in relieving intermittents, with the sulphate of quinine; and that as a fibrifuge and tonic it is capable of even a wider application than the latter agent.

We have used this remedy frequently in the treatment of fevers with very satisfactory results. It being comparatively cheap, we have often given it to charity cases.

We well remember the first case of remittent fever in which we used this drug. It was a well marked case of several days' standing in a negro man. We left twelve doses, four grains each, of the cincho-quinine, and directed him to take one every three hours continuously, until all were taken. No other remedy was used. Three or four days afterwards we met the patient on the street entirely recovered. He stated that the fever left him before he had taken all the powders. We could cite many cases of the successful use of this agent in intermittent fevers.

Dr. Akers, one of our subscribers, writes us that he has been recently experimenting with cincho-quinine, and reports an obstinate case of intermittent fever relieved by it. He gave thirty grains in two doses—one at 10 o'clock a. m., and another at 8 o'clock p. m.

We regard it, also, an excellent tonic. In a case of partial paralysis in a child three years old, the child being scarcely able to stand or walk, and attended with febrile exacerbations every evening, indicating the malarial source of the disease, we prescribed the following:

R.—Simple elixir ........................oz iv.
Cincho-quinine..........................dr. j.

M. S. Dose—a teaspoonful—give one every two hours during the forenoon. In three or four days the exacerbations were arrested. The remedy was afterward continued as a tonic three times a day for a period of three weeks, by which time the child wholly recovered. We think there is reason to believe that the cincho-quinine does contain all the essential properties of Peruvian bark, and that as a general tonic, especially in malarial complications, it is among the best in the materia medica.

### MEDICAL ASSOCIATION OF GEORGIA.

The present number of the Record goes to press too early for the proceedings of the State Medical Association. We will give a condensed account of them in our next issue.

Merrell, Thorp & Lloyd.—See new advertisement of the above house, on first advertising sheet. See also Wm. R. Warner & Cos.' new advertisement, and McKesson and Robbins. All first-class houses. Notice Bellevue Medical College advertisement. In short, read all our advertisements.

